# *Paracoccidioides brasiliensis* Isolated from Nine-Banded Armadillos (*Dasypus novemcinctus*) Reveal Population Structure and Admixture in the Amazon Basin

**DOI:** 10.3390/jof7010054

**Published:** 2021-01-15

**Authors:** Eduardo Bagagli, Daniel Ricardo Matute, Hans Garcia Garces, Bernardo Guerra Tenório, Adalberto Garcia Garces, Lucas Gomes de Brito Alves, Danielle Hamae Yamauchi, Marluce Francisca Hrycyk, Bridget Marie Barker, Marcus de Melo Teixeira

**Affiliations:** 1 Departamento de Microbiologia e Imunologia, Instituto de Biociências de Botucatu, Universidade Estadual Paulista/UNESP, Botucatu SP 18618-691, Brazil; eduardo.bagagli@unesp.br (E.B.); atiestorage@gmail.com (H.G.G.); atiweb@gmail.com (A.G.G.); lucasgbalves@gmail.com (D.H.Y.); 2 Department of Biology, University of North Carolina, Chapel Hill, NC 27599, USA; dmatute@email.unc.edu; 3 Faculdade de Medicina, Universidade de Brasília, Brasília DF 70910-900, Brazil; danihyamauchi@gmail.com (B.G.T.); bernardoguerra320@gmail.com (L.G.d.B.A.); 4 Faculdade de Ciências Biológicas e Agrárias, Universidade do Estado de Mato Grosso, Campus de Alta Floresta, Alta Floresta MT 78580-000, Brazil; marluce@unemat.br; 5 Pathogen and Microbiome Institute, Northern Arizona University, Flagstaff, AZ 86011, USA

**Keywords:** *Paracoccidioides*, Amazon, armadillos, paracoccidioidomycosis, admixture

## Abstract

Paracoccidioidomycosis is an endemic fungal disease to Latin America caused by at least five species-level genotypes of *Paracoccidioides*, named *P. lutzii*, *P. brasiliensis* (S1a and S1b populations), *P. americana*, *P. restrepiensis*, and *P. venezuelensis*. In this manuscript, we report on *Paracoccidioides* sp. sampling efforts in armadillos from two different areas in Brazil. We sequenced the genomes of seven *Paracoccidioides* isolates and used phylogenomics and populations genetics for genotyping. We found that *P. brasiliensis* and *P. lutzii* are both present in the Amazon region. Additionally, we identified two *Paracoccidioides* isolates that seem to be the result of admixture between divergent populations within *P. brasiliensis* sensu stricto. Both of these isolates were recovered from armadillos in a *P. lutzii* endemic area in Midwestern Brazil. Additionally, two isolates from human patients also show evidence of resulting from admixture. Our results suggest that the populations of *P. brasiliensis* sensu stricto exchange genes in nature. More generally, they suggest that population structure and admixture within species is an important source of variation for pathogenic fungi.

## 1. Introduction

Over 200 species of fungi have been reported to cause severe diseases in humans [1]. The Onygenalean fungi (Ascomycota, Eurotiomycetes) include a diverse array of endemic fungal pathogens that are tightly associated with many mammal species including humans. The fungi from these genera cause over 650,000 infections a year [2]. The infections caused by these fungi are most often triggered by the inhalation of infectious conidia or spores that undergo a dimorphic switch inside the lungs to yeast-like cells, provoking life-threatening diseases [3]. This morphological transformation is regulated by temperature, which has led to the hypothesis that the pathogens exists as mycelia in soil and as a yeast in animal reservoirs [4]. 

Among these fungi, the genus *Paracoccidioides* is of particular importance because it is almost exclusively diagnosed in Latin America and causes the most prevalent systemic mycosis in South America, paracoccidioidomycosis (PCM) [5]. The genus harbors five species, all of which are human pathogens [6,7,8,9,10,11]. Three of these species, *P. brasiliensis*, *P. americana*, and *P. lutzii*, are all widely distributed across the South American continent. The other two species, *P. restrepiensis* and *P. venezuelensis*, are restricted to Colombia and Venezuela, respectively [8,10,12,13]. The only species with extensive geographic sampling, *P. brasiliensis,* shows a strong population structure and harbors two distinct populations, named S1b and S1a, which have overlapping geographical occurrences in Southern Brazil, Argentina, and Paraguay [12]. *Paracoccidioides lutzii* is predominantly found in Amazon and Midwestern Brazil, and *P. americana* is found primarily in Southeastern Brazil, with a single report in Venezuela [8]. 

Dimorphic fungi have been isolated from soil and from mammal reservoirs. Armadillos, for example, are commonly animal reservoirs of *Paracoccidioides*. *Paracoccidioides brasiliensis* and *P. americana* have been isolated from *Dasypus novemcinctus* in Brazil [14,15,16,17], whereas *P. restrepiensis* was retrieved from *Cabassous centralis* specimens in Colombia [18]. DNA-based tests suggest the occurrence of these fungi in aerosols and soil samples obtained in the burrows of those mammals [14,19]. This association is not exclusive for *Paracoccidioides*; *Coccidioides* [20] and *Histoplasma* [21] have been isolated from armadillo tissues and their burrows. Collectively, these findings suggest that armadillos are a common reservoir for these pathogens [15].

In spite of the clear association between armadillos and *Paracoccidioides*, just a handful of isolates of this fungus obtained from armadillos have ever been sequenced. Three *P. brasiliensis*, one *P. americana* (São Paulo, Brazil), and one *P. restrepiensis* (Caldas, Colombia) isolates derived from armadillos had their genomes fully sequenced [13]. This paucity of genetic data from isolates from mammal reservoirs is particularly critical for the Amazon basin, one of the regions with the highest prevalence for PCM [5,22]. Here we aimed to bridge this gap by collecting *Paracoccidioides* isolates from armadillos from the Amazon and Southern Brazil and comparing their genotypes to previously sequenced genomes.

The genomes of the seven *Paracoccidioides* strains were sequenced, and we compared their genotypes to other animal and human-derived strains. Phylogenetic and population genetic analyses reveal that all the seven isolates belong to *Paracoccidioides brasiliensis* sensu stricto. We also found that two of these isolates are admixed and novel genotypes from S1a and S1b populations of *P. brasiliensis*. Our results suggest that intraspecific structure and admixture can be an important sources of variation in *P. brasiliensis* sensu stricto, and propose new mechanisms regarding the drivers of intraspecific variation in each of the species of *Paracoccidioides*. 

## 2. Materials and Methods 

### 2.1. Armadillos Capture, Euthanasia, and Fungal Isolation

**Figure 1:**
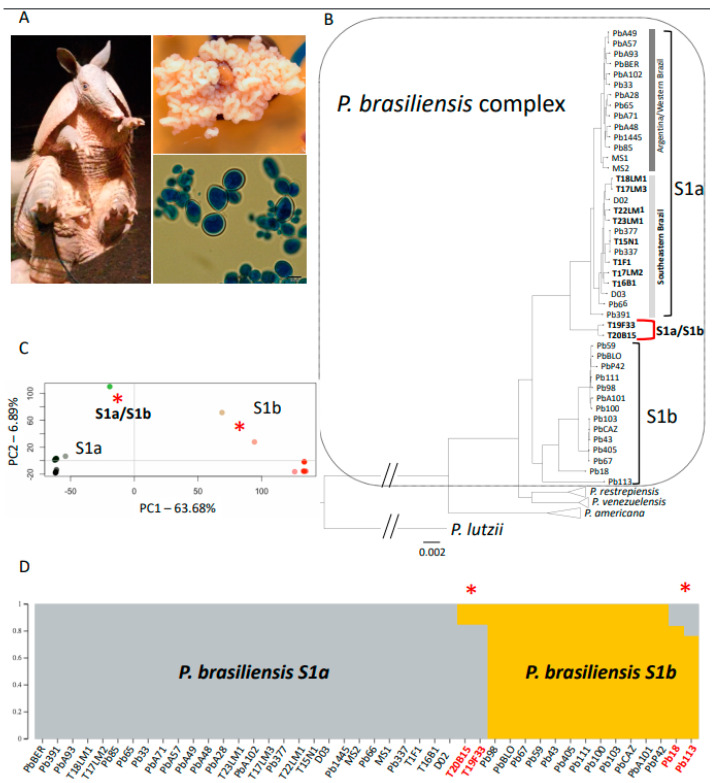
Genetic background of *Paracoccidioides brasiliensis* isolated from nine-banded armadillos in the Amazon basin. (**A**) Specimen collected, macroscopic characteristics of the fungal growth on tissue, and microscopic characteristics of yeast cells isolated from the tissue. (**B**) Genome-level maximum likelihood tree of 70 *Paracoccidioides* spp. genomes, indicating the placement of the Amazonian isolates T19F33 and T20B15 as a monophyletic group sister to *P. brasiliensis* S1a cluster. (**C**) Principal Component Analysis (PCA) reveals that *P. brasiliensis* S1a and S1b are populations separated along the PC1 axis corresponding to 63.68% of the total variation. (**D**) *ADMIXTURE* analysis of *P. brasiliensis* revealed admixed (*) S1a genotypes (T19F33 and T20B15) and S1b genotypes (Pb113 and Pb18). The proportion of admixture of each isolate is represented by height and colors of each of the two populations.

We captured six armadillos (*Dasypus novemcinctus*), four in São Paulo (Southeastern Brazil), and two in Mato Grosso (Midwestern, Brazil), using cage traps placed on the animal track or at the entrance of the armadillo’s burrow (Table 1). The permission for capturing and euthanizing the armadillos was obtained at the Instituto Brasileiro do Meio Ambiente e dos Recursos Naturais Renováveis, Instituto Chico Mendes (IBAMA/ICMBio—License number 30585-1) and at the Ethics Committee on the Use of Animals approved by the Biosciences Institute/UNESP (CEUA, protocol number 737, May 21th, 2015) in agreement with the National Council for the Control of Animal Experimentation, Brazilian Society of Science in Laboratory Animals (CONCEA) regulations. Hrycyk et al. describe the procedure in detail [19]. Briefly, the armadillos were submitted to euthanasia by injecting a combination of ZoletylR 50—Virbac, 0.2 mL/kg intramuscularly, as well as by cardiac puncture. Aseptically collected livers, spleens, and mesenteric lymph nodes were cleaned in 70% (*v/v*) alcohol, to remove unwanted contaminants and washed again in a sterile saline solution (0.9% *w/v*). Fragments ranging from 2 to 3 mm of the collected organs were cultivated in Mycosel Agar plates supplemented with gentamicin (50 μg/mL). The plates were incubated at 35 °C, for up to 45 days, and colonies with suspected morphology compatible with *Paracoccidioides* spp. were subcultured in Glucose Peptone Yeast Extract Agar (GPY). Seven armadillo-derived strains, five from Southeastern Brazil and two from Midwestern Brazil, were selected for whole genome sequencing, and the host characteristics, such as species, sex, weight, organ of isolation, and precise location, were collected (Figure 1A and Table 1).

### 2.2. DNA Extraction, Library Preparation and Genome Sequencing 

We used standard protocols for the extraction of DNA from fungal cells. Briefly, yeast cells of *Paracoccidioides* were disrupted using 0.5 mm beads and lysis buffer at the Precellys tissue homogenizer (Bertin Technologies, Rockville, MD, USA). Next, the DNA was extracted according to [19] and was quantified by using a Nanodrop 3300 fluorimeter stained with the PicoGreen dsDNA quantitation reagent (ThermoFisher, Waltham, MA, USA). About 1 μg of DNA was fragmented and used as input to prepare DNA paired-end libraries, using the KAPA HTP Library Preparation Kit. We used the KAPA Library Quantification Kit Illumina^®^ Platforms (Roche Sequencing Solutions, Pleasanton, CA, USA) to quantify the libraries via qPCR and then pooled them in a single Illumina flow cell. Sequencing was performed in an Illumina NextSeq 550 system (Illumina, San Diego, CA, USA), using a High Output Kit v2 (300 cycles), 2 × 150 bp. We used paired barcodes to de-multiplex reads, and individual read quality control was performed by using in-house methods.

### 2.3. Read Mapping and Single Nucleotide Polymorphism (SNP) Calling

We mapped the newly generated reads and reads from 63 previously sequenced *Paracoccidioides* isolates ([12], Supplementary Materials Table S1), to the *P. brasiliensis* Pb18 v2 assembly (ABKI00000000.2), using bwa v0.7.7 [23]. To identify Single Nucleotide Polymorphisms (SNPs), we used the NASP pipeline [24] after doing the following: (i) aligning trimmed and quality-filtered reads into the reference; (ii) re-aligning mapped reads to their corresponding reference genome via RealignerTargetCreator and IndelRealigner toolkits of GATK v3.3-0 to purge suspicious intervals; (iii) calling SNPs, using UnifiedGenotyper tool of GATK v3.3-0—the parameter “het” was set to 0.01; (iv) filtering resulting.vcf files, using the parameters QD = 2.0 || FS_filter = 60.0 || MQ_filter = 30.0 || MQ_Rank_Sum_filter = −12.5 || Read_Pos_Rank_Sum_filter = −8 [25,26]; and finally (v) removing SNPs with a coverage lower than 10X coverage across all samples or that were identified as being within duplicated regions in the reference by NUCmer [27].

### 2.4. Phylogenetic Analysis 

We used the SNP matrix to determine the phylogenetic placement of the *Paracoccidioides* spp. isolates obtained from armadillos. We did a Maximum Likelihood phylogenetic analysis with a total of 70 strains (63 previously sequenced and seven from this report). To generate the maximum likelihood tree, we loaded the SNP matrix into IQTREE v1.6.12 [28]. The best substitution SNP model was calculated by using the -m MFP function (ModelFinder) [29]. Since all the seven newly sequenced isolates we report here belong to *P. brasiliensis* sensu stricto (see Section 3), we rooted the tree with *P. lutzii*. To determine the support for the topology, we calculated support for each branch, using 1000 ultrafast bootstrap replicates coupled with a Shimodaira–Hasegawa-like approximate likelihood ratio test (SH-aLRT) [30,31]. 

### 2.5. Admixture Analyses

We studied whether any of the newly sequenced isolates showed evidence of being admixed. We used two complementary approaches. First, we visualized the arrangement of genetic variation within *P. brasiliensis* by using Principal Component Analysis (PCA). Similar analyses for the full *Paracoccidioides* genus can be found elsewhere [7,12]. We used the R package *adegenet* [32]. We only used biallelic sites extracted with the function *fasta2genlight*. To calculate the PCs, we used the function *glPca*. We restricted our analyses to the first two principal components (PCs), as they encompass most of the genetic variability (see Results). Second, we estimated the proportions of admixture in potentially admixed individuals, as revealed by the PCA. The phylogenetic tree revealed the existence of two populations (S1a and S1b; see Results and References [12,13]). We used *ADMIXTURE* [33] and conditioned the number of populations to be two (K = 2). This prior was based on the existence of the S1a and S1b populations. This analysis reveals the contribution of each of these two populations to the ancestry of each isolate within *P. brasiliensis* sensu stricto [34]. Please note that other *ADMIXTURE* scenarios might be more likely, but they do not serve to resolve the contributions of S1a and S1b to individual isolates. The proportion of admixture for each individual was plotted by using Structure Plot v2.0—http://omicsspeaks.com/strplot2/ [35]. 

## 3. Results

### 3.1. Genomic Data 

We obtained genomic data for seven *P. brasiliensis* isolates recovered from armadillos in two different endemic areas of PCM. The sequencing coverage ranged between 43.29× and 63.75× per site. Raw Illumina paired-end reads were deposited at the Sequence Read Archive (SRA), under the following deposit number SRR13267631- SRR13267637. After aligning the Illumina reads to the reference sequence and calling SNPs by using the GATK filters described in Reference [12], we generated a nucleotide matrix containing 831,476 polymorphic sites across all *Paracoccidioides* species. The matrix that included only strains from the *P. brasiliensis* species complex had 270,464 SNPs, which is consistent with the divergence between *P. lutzii* and the species from the *brasiliensis* complex. 

### 3.2. Phylogenetic Tree 

**Figure 2:**
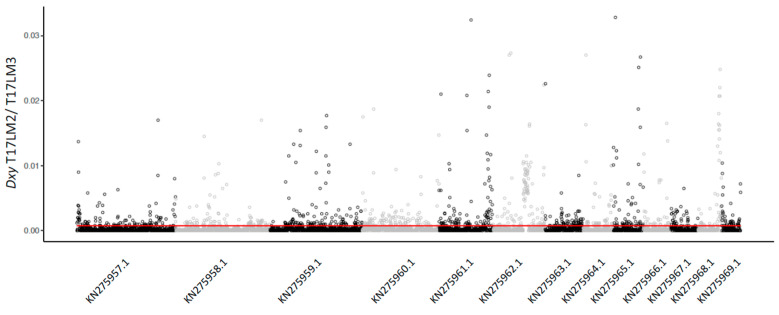
Whole-genome genetic distances between the isolates T17LM2 and T17LM3. Absolute genetic distance (dXY) between the isolates T17LM2 and T17LM3 were calculated by using 5 kb sliding window blocks and plotted across 13 largest scaffolds of the *P. brasiliensis* Pb18 reference genome.

Using the SNP matrix, we generated a maximum likelihood phylogenetic tree. As expected, a rooted phylogenetic tree with *P. lutzii* recovered all the four species within the *P. brasiliensis* species complex. Consistent with previous reports [12], we found that *P. brasiliensis* is formed by two structured populations previously deemed S1a and S1b. S1a is, in turn, composed of two subpopulations which follow a geographical pattern: One of the subpopulations is mostly found in Argentina and Eastern Brazil, while the other one is found in Southeastern Brazil. Our focus was to understand the genealogical relationships of the isolates collected from armadillos. The five isolates we recently collected from armadillos in Southwest Brazil and three previously collected isolates (T1F1, T15N1, and T16B1 [12,13]) appear within S1a and consistent with their geographical origin and are associated with the southeast subpopulation of *P. brasiliensis* sensu stricto. The isolates T22LM1, T23LM1, and T17LM2 were previously classified as S1b strains, using the *gp43* gene as a genetic marker [19], which highlights the potential for incorrect phylogenetic assignment when using a single locus as a proxy of ancestry. We obtained two distinct samples from a single armadillo (T17). While they are both assigned to the S1a population, they are not identical isolates. First, their position in the tree shows they are not each other’s closest relatives. For each of these two isolates, the closest identified relatives are other armadillo and clinical isolates. Second, a genome comparison shows alleles that are distinct, thus confirming they are not identical (Dxy = 7.38 × 10^−4^, Figure 2). The two of the armadillo isolates from the Amazon, T19F33 and T20B15, appear as a monophyletic group that is a sister clade to S1a, which might indicate the existence of an Amazon population within *P. brasiliensis* (Figure 1), a hypothesis we explore more deeply in the following section (see Section 3.3).

Most S1b isolates appear as part of two monophyletic groups. These two subpopulations broadly, but not perfectly, correspond to Argentina and Paraguay. Two isolates within S1b appear as deep branches with S1b and are not included in either of these two populations. Pb113 and, to a lesser extent, Pb18 (Figure 1B) appear to be lone branches within S1b. The phylogenetic signal from the T19F33/T20B15 dyad (classified in the tree as S1a) could be a true monophyletic clade, or it could be the result of conflicting phylogenetic signal in the genome. A similar occurrence could explain the phylogenetic position of Pb113 and Pb18 (classified in the tree as S1b). As a result of this observation, we explored the possibility of gene flow and patterns of admixture within *P. brasiliensis* sensu stricto.

### 3.3. Admixture

We investigated the partitioning of genetic variation within *P. brasiliensis* sensu stricto using genome-wide polymorphisms. Principal Component Analysis (PCA) biplots show that *P. brasiliensis* S1a and S1b are separated along the PC1 axis, which corresponds to 63.68% of the variance within the *P. brasiliensis* species (Figure 1C). Notably, the two S1a armadillo strains from the Amazon (T19F33 and T20B15) and Pb113 and Pb18 appear as intermediates between the two *P. brasiliensis* populations. Indeed, the distant phylogenetic position of these four isolates may be due to admixed ancestry of S1a and S1b. PC2 explains only ~5% of the total variance and seems to correspond to variants present in the Amazon armadillo strains. Next, we quantified the admixture proportions of these genetically intermediate isolates. As expected, when we forced *ADMIXTURE* to detect two groups within *P. brasiliensis* sensu stricto, we found that the two clusters correspond to S1a and S1b (Figure 1D). As suggested by the PCA, both Amazonian armadillo strains, T19F33 and T20B15, had evidence of admixed genotypes between the *P. brasiliensis* S1a and S1b. The proportion of admixture in both isolates was similar: S1a, 85%; S1b,15% (Figure 1D). Pb18 and Pb113 also show admixed genotypes (16 and 23% from S1a, respectively, and the rest from S1b). 

## 4. Discussion

Here we report our analysis of the genomes of seven isolates of *Paracoccidioides* isolated from six armadillos in the Amazon basin and Southern Brazil. Our results are relevant to three general aspects of the biology of *Paracoccidioides*: (i) the reinforcement of the close association of *Paracoccidioides* with armadillos and the potential role these animals play as an important reservoir for the fungus, (ii) the co-infection of a single armadillo with multiple isolates, and (iii) the extent of population structure and admixture within species of *Paracoccidioides*. We discuss each of these aspects as follows.

### 4.1. Paracoccidioides and Armadillos 

The association between armadillos and *Paracoccidioides* has been hypothesized since the 1960s [36] and confirmed in the 1980s [17]. So far, 140 armadillos (including six from the current work) have been euthanized by different research groups in six different states/endemic zones of the *Paracoccidioides*; 53 animals were positive and 87 were negative for fungal growth (Table S1). However, the association between armadillos and *Paracoccidioides* remains largely understudied. Three different species of *Paracoccidioides* have been isolated from two species of armadillos: *Dasypus novemcinctus* and *Cabassous centralis*. A single report detected DNA of Paracoccidioides sp. in tissue samples of a Dasypus septemcinctus road-killed specimen [37]. The most commonly isolated species from *D. novemcinctus* has been *P. brasiliensis* sensu stricto. Out of the 39 isolates that have been obtained and genotyped from *D. novemcinctus*, 37 belong to *P. brasiliensis* sensu stricto, and most of the strains genotyped by whole genome analysis fit into the population S1a (Supplementary Materials Table S2, [9,12,13,19]). Whether this pattern is the result of limited sampling effort and sample size, focused in localities where S1a is endemic, or whether S1a is more likely to infect armadillos, will remain unknown until a comprehensive sampling of these mammals is completed across the endemic regions. *Paracoccidioides americana* has been isolated from *Dasypus novemcinctus* on two occasions (T10 and T18; Table 1, [9,19]). *Paracoccidioides restrepiensis* has been isolated from two different species: *D. novemcinctus* [38] and *C. centralis* [18]. This latter species of armadillo is restricted to the northern part of South America and Central America, and the only species of *Paracoccidioides* that overlaps with C. centralis’ range is *P. restrepiensis*. The two other species of *Paracoccidioides* that have not been isolated from armadillos are *P. lutzii* and *P. venezuelensis*. Both species remain sparsely sampled and it is premature to conclude whether or not they infect armadillos.

An unknown aspect of the relationship between armadillos and fungal pathogens, and in particular of *Paracoccidioides*, is whether additional species of armadillos, besides *D. novemcinctus* and *C. centralis*, are susceptible to fungal infections. The genera *Dasypus* and *Cabassous*, the two known armadillo reservoirs of *Paracoccidioides*, diverged approximately 45 million years ago. *Dasypus*, the only genus within the family Dasypodidae, contains seven extant species; *Cabassous* belongs to the family Chlamyphoridae, which harbors 14 extant species. Whether or not the other 21 armadillo species are infected with *Paracoccidioides*, or with other fungi, remains an open question. PCR surveys in autopsied tissues suggest the presence of *Paracoccidioides* in other armadillo species, but, to date, no isolate has been obtained (Supplementary Materials Table S1, [37]). There is some evidence that armadillos are not the only animal reservoir of *Paracoccidioides*. For example, *Paracoccidioides* infections are not rare in dogs [39,40]. Roadkill samples of *Didelphis albiventris*, *Gallictis vittata*, *Procyon cancrivorus*, and *Sphiggurus spinosus* tested positive for *Paracoccidioides* DNA [37]. A southern two-toed sloth captured in French Guiana showed signs consistent with a PCM infection [41]. Anti-*P. lutzii* and anti-*P. brasiliensis* antibodies have been reported in wild and domestic animals in Southern Brazil, suggesting that natural infections of both species might occur [42]. The co-evolutionary association of these organisms remains an underdeveloped area of medical mycology and other infectious diseases. In general, the ecology and life history of *Paracoccidioides* remain largely unknown, and a concerted community effort will be required to identify ecological features important for the distribution of each of the species in the genus.

### 4.2. Coinfection by Multiple Isolates 

One of the armadillos (T17) showed coinfection by two different isolates of *P. brasiliensis* (Figure 1B). Coinfections suggest the potential of close contact within animal reservoirs and have two major implications. First, they suggest the possibility of recombination within animal reservoirs, a phenomenon observed in *Candida albicans* [43]. Alternatively, different isolates could exclude each other and be partitioned in different tissues or niches. Interestingly, at least one armadillo was confirmed to be co-infected with *P. americana* and *P. brasiliensis* simultaneously (sample T18, [19]). This co-infection is prima facie evidence that *P. brasiliensis* sensu stricto and *P. americana* share an ecological niche, have the opportunity to interbreed, and yet they show no evidence of gene exchange. Because little is known about gene exchange and frequency of sexual recombination in these organisms, the identification of barriers to gene flow are not currently known.

### 4.3. Population Structure and Admixture 

The advent of genomics has revealed that species and populations hybridize and exchange genes much more than previously thought. In the case of *Paracoccidioides*, there has been an emphasis to determine whether different species in the genus hybridize and exchange genes [7,44]. While recently diverged species (e.g., *P. restrepiensis* vs. *P. venezuelensis*) show some minimal evidence for interspecific admixture, the most divergent species (e.g., *P. americana* and any other species) show no evidence of interspecific gene flow [44]. Other fungal pathogens from the same family also show some evidence of gene exchange between species [45,46]. In this report, we identified four isolates with evidence of admixed ancestry from structured populations within *P. brasiliensis* sensu stricto. Admixtures are now known to be much more common than previously thought [12]. The possibility of hybridization and admixture generating allelic combinations that can be selected upon will depend on the density of alleles that increased fitness in the recipient population, the density of hybrid incompatibilities, and the recombination rates between positively and negatively selected alleles [47,48,49]. In this report, we focused on the magnitude of gene exchange between populations of the most-extensively sampled species of *Paracoccidioides* and *P. brasiliensis* sensu stricto. Interspecific hybrids can serve as a bridge for gene exchange, but their fitness is often reduced because of hybrid incompatibilities. Admixed individuals from different populations, but from the same species, are less likely to show fitness defects (because there is lower chances of hybrid incompatibility [50,51,52]) and admixture is more likely to generate allelic combinations that can be favored by selection [53]. The genomes of isolates from Amazonian armadillos revealed that populations within *P. brasiliensis* interbreed and exchange genes. Moreover, clinical isolates from Southern Brazil also have admixed origin between the same populations, suggesting that the admixture between these two populations might occur over a large geographical scale. Alternatively, it might be that admixture only occurs in a single location, but there has been migration of admixed genotypes. Since patients are more likely than armadillos to move thousands of miles, if there has been movement, it is more likely that the admixture occurred in the Amazon. 

## 5. Conclusions

Studies on *Paracoccidioides* have focused on the partition of genetic diversity among cryptic species. The last fifteen years have seen the identification of five previously undescribed species within the genus [7,11]. This robust taxonomic classification, which includes morphological traits and molecular markers to identify *Paracoccidioides* species, should be the launching point in defining processes that drive the evolution of *Paracoccidioides* species and populations. In this report, we showed that population structure and intraspecific admixture might be important contributors to genetic diversity within fungal species. Our results, and others previously published [14,15,19], have revealed that armadillos are commonly infected by *P. brasiliensis*, *P. americana*, and *P. restrepiensis*, but not by *P. lutzii*. As of now, the only reliable isolate retrieval mechanism is to sacrifice the animal or to collect them postmortem (e.g., roadkill, [37]). The development of non-lethal sampling would increase the possibility of recovering isolates at much higher rates, and thus improve our understanding of the basic biology, genetics, and ecology of the fungus. Only denser sampling of isolates and a precise quantification of their levels of admixture will reveal the true biogeographic structure of intraspecific allele exchange within *P. brasiliensis* sensu stricto.

## Figures and Tables

**Table 1:** Strain, host, sex, host weight, location, geographical coordinates, organ, and genotypes for each of the strains analyzed by whole genome sequencing.

Strain	Host	Sex	Host Weight (kg)	Geographic Area	Coordinates *	Organ	Genotype
T17LM2	*D. novemcinctus*	male	4.7	Southeast, SP (Botucatu)	22°48′02″ S/48°23′24″ W	Mesenteric Lymph node	S1a
T17LM3	*D. novemcinctus*	male	4.7	Southeast, SP (Botucatu)	22°48′02″ S/48°23′24″ W	Mesenteric Lymph node	S1a
T18LM1	*D. novemcinctus*	male	3.9	Southeast, SP (Botucatu)	22°48′02″ S/48°23′24″ W	Mesenteric Lymph node	S1a
T22LM1	*D. novemcinctus*	female	3.9	Southeast, SP (São Manoel)	22°34′16″ S/48°25′18″ W	Mesenteric Lymph node	S1a
T23LM1	*D. novemcinctus*	male	4.3	Southeast, SP (São Manoel)	22°34′16″ S/48°25′18″ W	Mesenteric Lymph node	S1a
T19F33	*D. novemcinctus*	male	6.0	Midwest, MT (Alta Floresta)	10°04′23″ S/56°09′34″ W	Liver	S1a/S1b
T20B15	*D. novemcinctus*	male	6.0	Midwest, MT (Alta Floresta)	10°04′23″ S/56°09′34″ W	Spleen	S1a/S1b

* Coordinates by Universal Transverse Mercator (UTM).

## Data Availability

Genomic data supporting the herein reported results can be found at BioProject PRJNA685988.

## Supplementary Materials

The following are available online at https://www.mdpi.com/2309-608X/7/1/54/s1. Table S1: Species of armadillo, number of individuals sampled, and positive and negative animals tested so far for the isolations of *Paracoccidioides* spp. Table S2: *Paracoccidioides* sp. armadillos isolate name and species diagnosed. References [54,55,56] were cited in the Supplementary Materials.
